# Protein kinase Hsl1 phosphorylates Pah1 to inhibit phosphatidate phosphatase activity and regulate lipid synthesis in *Saccharomyces cerevisiae*

**DOI:** 10.1016/j.jbc.2024.107572

**Published:** 2024-07-14

**Authors:** Shoily Khondker, Gil-Soo Han, George M. Carman

**Affiliations:** Department of Food Science and the Rutgers Center for Lipid Research, Rutgers University, New Brunswick, New Jersey, USA

**Keywords:** lipid, phospholipid, triacylglycerol, Pah1 phosphatidate phosphatase, phosphatidate, diacylglycerol, Hsl1 protein kinase, phosphorylation, yeast

## Abstract

In *Saccharomyces cerevisiae*, Pah1 phosphatidate (PA) phosphatase, which catalyzes the Mg^2+^-dependent dephosphorylation of PA to produce diacylglycerol, plays a key role in utilizing PA for the synthesis of the neutral lipid triacylglycerol and thereby controlling the PA-derived membrane phospholipids. The enzyme function is controlled by its subcellular location as regulated by phosphorylation and dephosphorylation. Pah1 is initially inactivated in the cytosol through phosphorylation by multiple protein kinases and then activated *via* its recruitment and dephosphorylation by the protein phosphatase Nem1-Spo7 at the nuclear/endoplasmic reticulum membrane where the PA phosphatase reaction occurs. Many of the protein kinases that phosphorylate Pah1 have yet to be characterized with the identification of the target residues. Here, we established Pah1 as a *bona fide* substrate of septin-associated Hsl1, a protein kinase involved in mitotic morphogenesis checkpoint signaling. The Hsl1 activity on Pah1 was dependent on reaction time and the amounts of protein kinase, Pah1, and ATP. The Hsl1 phosphorylation of Pah1 occurred on Ser-748 and Ser-773, and the phosphorylated protein exhibited a 5-fold reduction in PA phosphatase catalytic efficiency. Analysis of cells expressing the S748A and S773A mutant forms of Pah1 indicated that Hsl1-mediated phosphorylation of Pah1 promotes membrane phospholipid synthesis at the expense of triacylglycerol, and ensures the dependence of Pah1 function on the Nem1-Spo7 protein phosphatase. This work advances the understanding of how Hsl1 facilitates membrane phospholipid synthesis through the phosphorylation-mediated regulation of Pah1.

Phosphatidate (PA) is a minor membrane phospholipid that plays multiple roles in the lipid synthesis of yeast and higher eukaryotic organisms (1, 2, 3) (Fig. 1). In the yeast *Saccharomyces cerevisiae*, PA serves as a common precursor for the synthesis of all membrane phospholipids *via* CDP-diacylglycerol (CDP-DAG) and for the synthesis of the storage lipid triacylglycerol (TAG) *via* diacylglycerol (DAG) (3, 4, 5, 6) (Fig. 1). Mutants defective in the synthesis of phosphatidylcholine and phosphatidylethanolamine *via* the CDP-DAG pathway (7, 8, 9, 10, 11, 12, 13, 14) require the synthesis of these phospholipids from DAG *via* the CDP-choline (15, 16, 17, 18) and CDP-ethanolamine (19, 20, 21, 22) branches of the Kennedy pathway by supplementation of choline and ethanolamine, respectively (6, 23, 24, 25) (Fig. 1). The utilization of PA for the synthesis of the diverse lipids is governed by nutrient availability, growth phase, and gene mutations (3, 5, 6). For example, PA is primarily utilized for phospholipid synthesis *via* CDP-DAG when cells actively grow, but it is mainly utilized for TAG synthesis *via* DAG when the cells progress into the stationary phase (4, 26). In addition to its use as a lipid precursor, PA plays a major role in the transcriptional regulation of UAS_INO_-containing phospholipid synthesis genes *via* the Henry (Opi1/Ino2-Ino4) regulatory circuit by sequestering, in concert with Scs2, the Opi1 repressor at the nuclear/ER membrane (3, 5, 6, 27, 28, 29).

**Figure 1:**
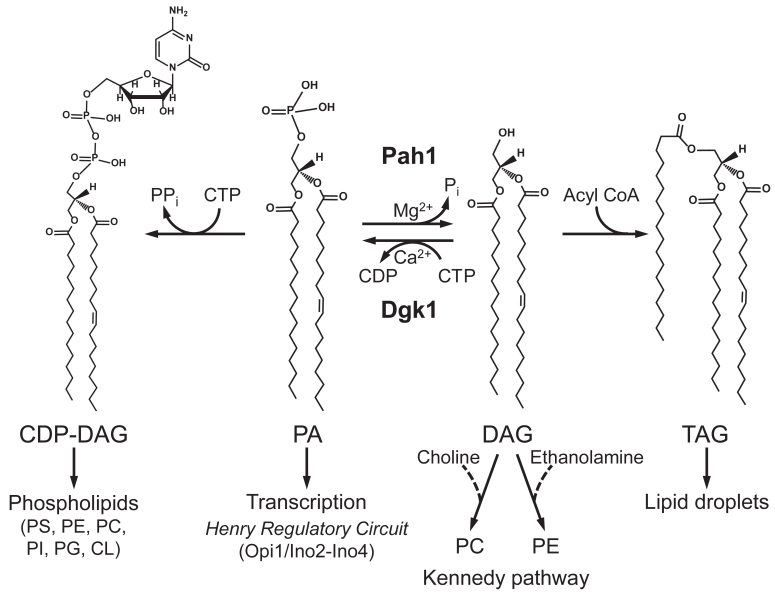
**Roles of Pah1 in lipid synthesis.** The structures of CDP-DAG, PA, DAG, and TAG are shown with fatty acyl groups of 16 and 18 carbons with and without a single double bond where indicated. Pah1 plays a key role in the use of PA for the synthesis of membrane phospholipids *via* CDP-DAG or the synthesis of TAG *via* DAG. The PAP reaction is counterbalanced by the CTP-dependent conversion of DAG to PA by the diacylglycerol kinase Dgk1. The DAG produced by the PAP reaction may be used for the synthesis of phosphatidylcholine and phosphatidylethanolamine *via* the CDP-choline and CDP-ethanolamine branches, respectively, of the Kennedy pathway when cells are supplemented with choline and/or ethanolamine. In addition to its role in lipid synthesis, PA signals the transcriptional regulation of phospholipid synthesis genes *via* the Henry regulatory circuit. More comprehensive pathways of lipid synthesis, along with details of the Henry regulatory circuit may be found in Refs. (5, 6). CL, cardiolipin; PC, phosphatidylcholine; PE, phosphatidylethanolamine; PG, phosphatidylglycerol; PI, phosphatidylinositol; PS, phosphatidylserine.

Among the enzymes that control the metabolism of PA (2, 3, 6), the *PAH1*-encoded PA phosphatase (PAP) (30) has emerged as a key regulator of PA levels and its utilization for lipid synthesis (3, 5, 31, 32) (Fig. 1). PAP catalyzes the Mg^2+^-dependent dephosphorylation of PA to produce DAG (30) (Fig. 1). The expression of Pah1 is low during the exponential phase when cells actively divide and PA is needed for the synthesis of membrane phospholipids, but increased as cells progress into the stationary phase when energy storage takes priority over cell division and PA is increasingly used for the synthesis of TAG (5, 6, 33). The physiological importance of Pah1 is highlighted by numerous studies that have examined the effects of the *pah1*Δ mutation on lipid synthesis and cell physiology (3, 30, 34, 35, 36, 37, 38, 39, 40, 41, 42, 43, 44, 45, 46). For example, loss of PAP activity causes a drastic decrease in TAG synthesis with a concomitant increase in phospholipid synthesis, as well as a diverse set of deleterious phenotypes that include the aberrant expansion of the nuclear/ER membrane and a shortened chronological life span with apoptotic cell death in the stationary phase (3, 30, 34, 35, 36, 37, 38, 39, 40, 41, 42, 43, 44, 45, 46).

Pah1 is a peripheral membrane enzyme and its PAP function occurs at the nuclear/endoplasmic reticulum (ER) membrane surface (30, 47, 48). The subcellular localization of Pah1 is controlled by the posttranslational modifications of phosphorylation and dephosphorylation (49). Pah1 is phosphorylated by multiple protein kinases (50, 51, 52, 53, 54, 55, 56) (Fig 2*A*), and the phosphorylated enzyme is localized to the cytosol (50, 57). Moreover, phosphorylated Pah1 is protected against proteasomal degradation (58, 59). Some of its phosphosites (*e.g.*, Ser-10, Ser-511, and Ser-814) are unique to specific protein kinases while others (*e.g.*, Ser-602, Ser-677, Ser-748, and Ser-773) are common to different protein kinases (49) (Fig. 2*A*). Some phosphorylations are hierarchical in nature, where the phosphorylation at one site affects the phosphorylation at another site (49, 56). Additionally, phosphorylations of Pah1 by some protein kinases stimulate (*e.g.*, casein kinase I) or inhibit (*e.g.*, Pho85 and Rim11) its PAP activity (50, 55, 56) (Fig. 2*B*).

**Figure 2:**
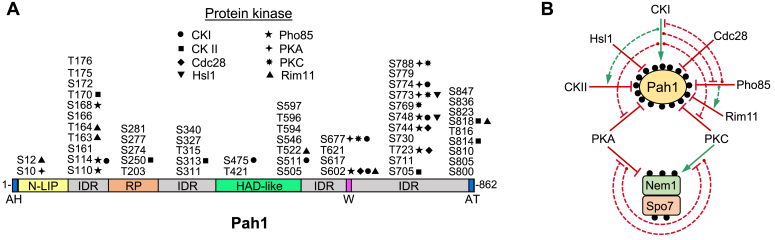
**Pah1 domains, regions, and phosphorylation sites; impacts and interrelationships of phosphorylation and dephosphorylation on Pah1 function.***A*, the schematic shows the domains/regions of Pah1. The N-LIP and the haloacid dehalogenase (HAD)-like domains are required for PAP activity (30, 41). The N-terminal amphipathic helix (*AH*) is responsible for interaction with the membrane (48). The regulation of the phosphorylation (RP) domain facilitates phosphorylation (93). The essential tryptophan contained within the WRDPLVDID domain, which is C-terminal to the HAD-like domain (138), is required for the *in vivo* catalytic function (89, 138). The acidic tail (*AT*) associates with the Nem1-Spo7 complex (62) through ionic interaction with the C-terminal basic tail of Spo7 (139). The intrinsically disordered regions (IDRs) contain almost all of the sites of phosphorylation that serve for interaction with the Nem1-Spo7 complex. The serine (*S*) and threonine (*T*) residues known to be phosphorylated (50, 51, 52, 53, 54, 55, 56, 57, 75, 83, 84, 89, 140, 141, 142, 143, 144, 145, 146, 147) are grouped at their approximate regions in the Pah1 protein. The sites phosphorylated by casein kinase I (*CKI*) (55), casein kinase II (*CKII*) (54), Cdc28 (51), Hsl1 (this study), Pho85 (50), protein kinase A (*PKA*) (52), protein kinase C (*PKC*) (53), and Rim11 (56) are indicated. *B*, the positive (*solid green arrow*) or negative (*solid blunted red line*) impacts of Pah1 phosphorylation (denoted by the small *black circles*) by the indicated protein kinases and Pah1 dephosphorylation by Nem1-Spo7, whose components are also subject to phosphorylation by protein kinases A and C (65, 66), are indicated. The casein kinase I phosphorylation of Pah1 stimulates its subsequent phosphorylation by casein kinase II (*dashed green arrow*) but inhibits its subsequent phosphorylations by Pho85, proteins A and C (*dashed blunted red lines*) (55). Pho85 phosphorylation of Pah1 stimulates its subsequent phosphorylation by Rim11 (*dashed green arrow*) (56) but inhibits its subsequent phosphorylation by casein kinase I (*dashed blunted red line*) (55). The phosphorylations of Nem1-Spo7 by protein kinase A inhibits phosphorylation of Spo7 by protein kinase C, whereas the phosphorylation of the complex by protein kinase C inhibits phosphorylation of Nem1 by protein kinase A (*dashed blunted red lines*) (65, 66).

In contrast to Pah1 phosphorylation, its dephosphorylation is catalyzed by a single protein phosphatase complex that is composed of the Nem1 (catalytic) and Spo7 (regulatory) subunits (34, 57, 60, 61) (Fig. 2*B*). Nem1-Spo7 has the function of activating Pah1; it recruits Pah1 to the nuclear/ER membrane and dephosphorylates the enzyme (34, 48, 49, 57, 61, 62). The dephosphorylation permits Pah1 to hop onto and scoot along the membrane to recognize its substrate PA and catalyze the PAP reaction (63). In a feedforward mechanism, PA stimulates Nem1-Spo7 phosphatase activity (64). Additionally, the dephosphorylation of Pah1 stimulates its PAP activity (61). Like Pah1, the Nem1 and Spo7 subunits are subject to phosphorylation by protein kinases A and C (65, 66) (Fig. 2*B*). The phosphorylations of the phosphatase complex by these protein kinases have opposing effects on Pah1 activity and its cellular function such as TAG synthesis; protein kinase A inhibits the protein phosphatase (65), whereas protein kinase C stimulates the enzyme (66). Consistent with the requirement of the phosphatase complex for Pah1 localization and PAP activity, the *nem1*Δ or *spo7*Δ mutation elicits the same phenotypes characteristic of those caused by the *pah1*Δ mutation (60, 67, 68).

Phosphoproteomics studies have shown that Pah1 is phosphorylated at 56 sites (Fig. 2*A*) and a substrate for ∼ 20 protein kinases (49, 69). The protein kinases that have been characterized for Pah1 phosphorylation with the identification of target sites include the cyclin-dependent kinases Cdc28 and Pho85, casein kinases I and II, protein kinases A and C, and the glycogen synthase kinase 3β homolog Rim11 (50, 51, 52, 53, 54, 55, 56) (Fig. 2*A*). This information along with analyses of cells bearing Pah1 deficient in phosphorylation at the kinase-specific sites has provided insight into the role of each protein kinase in the regulation of Pah1 localization, catalytic activity, and stability (49).

Many of the protein kinases that phosphorylate Pah1 have yet to be characterized and their target sites of phosphorylation need to be defined. In this study, we focused on the septin-associated protein kinase Hsl1 (histone synthetic lethal1), one of the kinases shown to phosphorylate Pah1 in the global analysis of yeast protein phosphorylation (69). Hsl1 is involved in the *S. cerevisiae* morphogenesis checkpoint, a mechanism that ensures the presence of a bud prior to progression through the mitotic phase of the cell cycle (70, 71, 72). As the morphogenesis checkpoint function is relevant to the exponential growth during which Pah1 is highly phosphorylated (49), we hypothesized that the Hsl1 phosphorylation of Pah1 would have an inhibitory effect on PAP activity and promote the synthesis of membrane phospholipids. Here, we established that Pah1 is a *bona fide* substrate of Hsl1 and that its phosphorylation on Ser-748 and Ser-773 has an inhibitory effect on PAP activity. Mutational analysis of Ser-748 and Ser-773 indicated that the Hsl1-mediated phosphorylation at these sites facilitates membrane phospholipid synthesis at the expense of triacylglycerol, and ensures the dependence of Pah1 function on the Nem1-Spo7 protein phosphatase.

## Results

### Growth-dependent expression and purification of Hsl1

**Figure 3:**
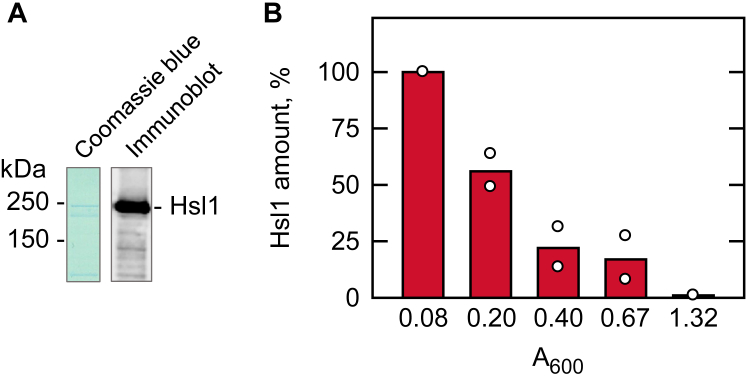
**Isolation of Hsl1 and growth-dependent expression of the protein.***A*, the isolated Hsl1 was subjected to SDS-PAGE (10% polyacrylamide gel) and stained with Coomassie blue (*left*). The enzyme preparation was also subjected to immunoblot analysis using calmodulin-binding peptide antibody (*right*). The positions of molecular mass standards and Hsl1 are indicated. The Hsl1 protein from a replicate gel was used for proteolytic digestion and phosphopeptide identification by LC-MS/MS analysis (Table S1). *B*, cells expressing Hsl1 were grown to the indicated cell densities (A_600_) in the SC medium. Lysates equivalent to 0.2 A_600_ units of cells were used for immunoblot analysis with anti-calmodulin binding peptide antibody. The amount of Hsl1-TAP was quantified with the ImageQuant software. The data shown is the average of two independent experiments with individual data points shown.

Hsl1 fused with a TAP tag at the C-terminus was isolated from the extracts of early exponential-phase cells by affinity chromatography with IgG-Sepharose. Since the TAP tag was resistant to cleavage by TEV protease, the protein kinase was purified without removing the affinity tag. Hsl1 was expressed at a low level and easily degraded, and the yield of its purification was ∼ 1 μg per ml (Fig 3*A*). The identity of Hsl1-TAP migrating at the estimated molecular mass of ∼190 kDa was confirmed by immunoblotting with an antibody against calmodulin-binding peptide of the affinity tag (73, 74) (Fig. 3*A*) as well as by LC-MS/MS analysis of the fusion protein-derived peptides (61% coverage of the protein) (Table S1).

The effect of growth on the level of Hsl1 was examined to better understand the role of the protein kinase in the regulation of Pah1. The *S. cerevisiae* cells expressing the TAP-tagged chromosomal *HSL1* were grown in an SC medium and analyzed for the level of Hsl1-TAP by immunoblotting with an anti-calmodulin-binding peptide antibody. During cell growth, the cellular level of the protein kinase was highest at the early exponential phase (A_600_ = 0.08) and decreased to the undetectable at the late exponential phase (A_600_ = 1.32) (Fig. 3*B*).

### Hsl1 phosphorylates Pah1 on Ser-748 and Ser-773

**Figure 4:**
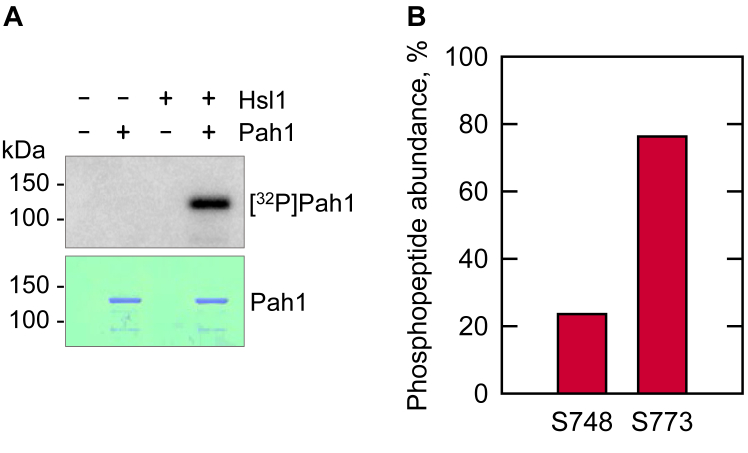
**Hsl1 phosphorylates Pah1 on Ser-748 and Ser-773.***A*, 1 μg Pah1 was incubated for 10 min at 30 °C with 100 μM [γ-^32^P]ATP (3000 cpm/pmol) in the absence (−) and presence (*+*) of 5 ng Hsl1. The reaction mixtures were resolved by SDS-PAGE (10% polyacrylamide gel) and subjected to protein staining with Coomassie blue (*lower*), followed by phosphorimaging (*upper*). The data shown are representative of three experiments. *B*, Pah1 phosphorylated by Hsl1 was extracted from an SDS-polyacrylamide gel, reduced, and alkylated, followed by digestion with trypsin; the resulting peptides were analyzed by LC-MS/MS. The abundance of phosphopeptides containing the indicated phosphorylation sites was estimated from intensities reported by Proteome Discoverer and expressed as a percentage of the intensities of all phosphopeptides identified for the protein (Table S1). Shown are only the phosphorylation sites that are confidently assigned at ≥ 1% of the total phosphopeptide abundances.

To examine the phosphorylation of Pah1 by the purified Hsl1, we utilized the His-tagged Pah1 purified from heterologous expression in *Escherichia coli* (30). The Hsl1 protein kinase isolated from yeast maintains its endogenous phosphorylation (75, 76, 77, 78, 79, 80, 81, 82, 83, 84, 85, 86), some of which is required for its function (87, 88). By using Pah1 expressed in *E. coli*, we examined its phosphorylation in the absence of the endogenous phosphorylation that occurs in yeast (57, 89). The Hsl1 activity on Pah1 was shown by following the incorporation of the radioactive phosphate from [γ-^32^P]ATP into the protein. In the radioactive assay, the ^32^P-labeled Pah1 was resolved from [γ-^32^P]ATP by SDS-PAGE, and radioactive Pah1 was then quantified by phosphorimaging (Fig. 4*A*). In a parallel assay, Pah1 was phosphorylated by Hsl1 with non-radioactive ATP and resolved by SDS-PAGE. The polyacrylamide gel slice containing phosphorylated Pah1 was subjected to proteolytic digestion and analyzed by LC-MS/MS (Fig. 4*B* and Table S1). Based on the abundance of phosphopeptides, Ser-748 (24%) and Ser-773 (76%) were the major sites phosphorylated by Hsl1.

### Characterization of Hsl1 activity on Pah1

**Figure 5:**
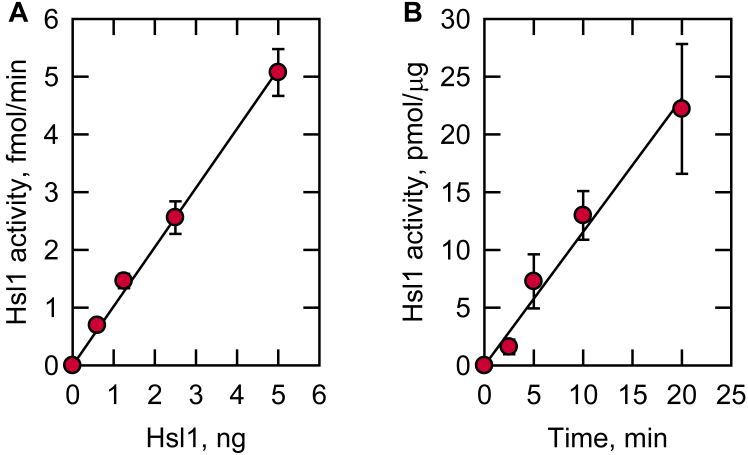
**Hsl1 protein kinase activity on Pah1 is dependent on the amount of Hsl1 and the reaction time.** Pah1 was incubated at 30 °C with Hsl1 and [γ-^32^P]ATP, subjected to SDS-PAGE, and analyzed by phosphorimaging. The enzyme assay was conducted by varying the amount of Hsl1 (*A*) and the reaction time (*B*). *A*, 500 nM Pah1/100 μM ATP/10 min; *B*, 500 nM Pah1/100 μM ATP/5 ng Hsl1. The data shown are means ± S.D. (*error bars*) from triplicate assays.

**Figure 6:**
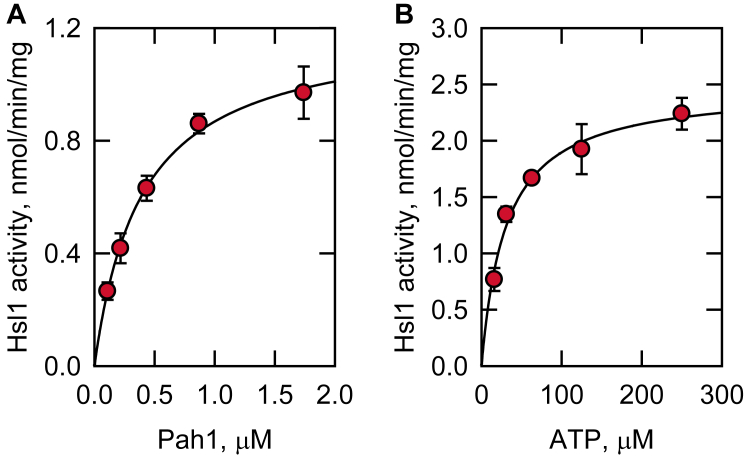
**Hsl1 protein kinase activity on Pah1 is dependent on the amounts of Pah1 and ATP.** Pah1 was incubated at 30 °C with Hsl1 and [γ-^32^P]ATP, subjected to SDS-PAGE, and analyzed by phosphorimaging. The enzyme reaction was conducted by varying the amounts of Pah1 (*A*), and ATP (*B*). *A*, 100 μM ATP/5 ng Hsl1/10 min; *B*, 500 nM Pah1/5 ng Hsl1/10 min. The data shown are means ± S.D. (*error bars*) from triplicate assays.

The kinase activity of Hsl1 was characterized by Pah1 as a substrate. The extent of Pah1 phosphorylation was dependent on the amount of Hsl1 and time of reaction (Fig. 5*A* and *B*, respectively), indicating that the protein kinase activity follows zero-order kinetics. In addition, the Hsl1 activity was dependent on the concentrations of Pah1 and ATP (Fig. 6*A* and *B*, respectively). The *V*_max_ and *K*_*m*_ values of Hsl1 protein kinase with respect to Pah1 were 1.2 nmol/min/mg and 0.4 μM, respectively, and those for ATP were 2.5 nmol/min/mg and 31 μM, respectively. Overall, these enzymological properties support the conclusion that Pah1 is a *bona fide* substrate of the Hsl1 protein kinase.

### Hsl1 phosphorylation of Pah1 inhibits its PAP activity

**Figure 7:**
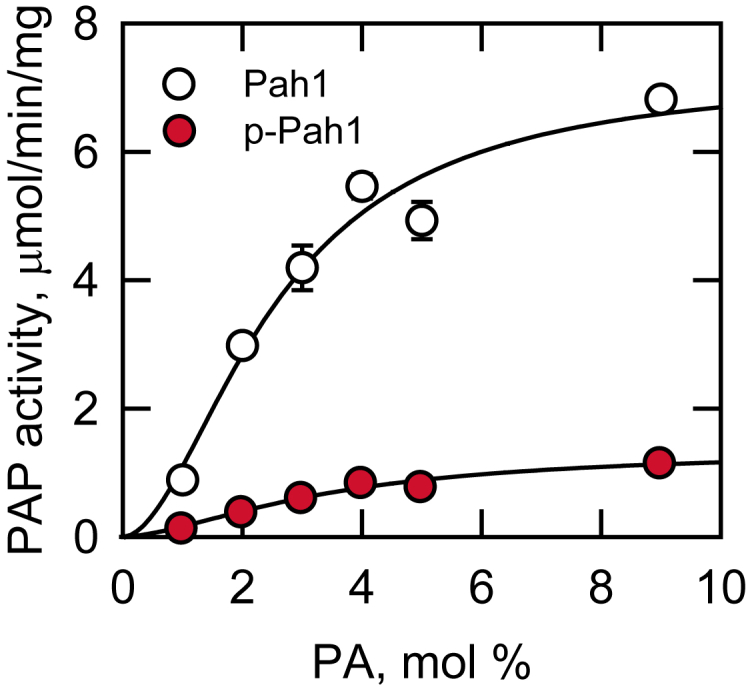
**Hsl 1 phosphorylation of Pah1 inhibits its PAP activity.** 1 μg Pah1 was phosphorylated by 5 ng Hsl1 for 2 h with 100 μM ATP in a total volume of 20 μl. The unphosphorylated Pah1 as control was incubated under the same reaction condition in the absence of Hsl1. After the incubation, 10% of the reaction mixture was measured for PAP activity. The surface concentration of PA (mol %) was adjusted by maintaining the molar concentration of PA at 0.2 mM and varying the molar concentration of Triton X-100 (90). The data shown are means ± S.D. (*error bars*) from triplicate assays. *p-Pah1*, phosphorylated Pah1.

The phosphorylation of Pah1 by Hsl1 was examined for its effect on PAP activity. Immediately after its phosphorylation, Pah1 was measured for PAP activity by following the production of water-soluble ^32^P_i_ from chloroform-soluble ^32^P_i_-labeled PA in the Triton X-100/PA-mixed micelles (30, 90). Under this assay condition, PAP activity is dependent on the surface concentration of PA, but independent of its molar concentration (90). The phosphorylation of Pah1 caused a decrease in the catalytic efficiency of PAP activity; the *V*_max_ of the phosphorylated enzyme (1.4 μmol/min/mg) was 5-fold lower than the unphosphorylated enzyme (7.2 μmol/min/mg) (Fig. 7). The *K*_m_ value and Hill number for PA of the phosphorylated (3.5 mol% and *n* = 1.6, respectively) and unphosphorylated (2.5 mol% and *n* = 1.8, respectively) forms of Pah1 were not significantly different.

### Hsl1 contributes to the Nem1-Spo7-mediated regulation of Pah1

The cellular effects of the Hsl1-mediated phosphorylation of Pah1 were examined by the analysis of cells expressing phosphorylation-deficient mutant forms of the enzyme. The mutant alleles of *PAH1* (S748A, S773A, and S748A/S773A) were expressed on a low-copy plasmid in the *pah1*Δ and *pah1*Δ *nem1*Δ strains. The *pah1*Δ *nem1*Δ mutant, which lacks the Nem1 catalytic subunit (34, 60), was used to examine the dependency of the Pah1 function on Nem1-Spo7 (51). The *nem1*Δ mutation also affords the analysis of the phosphorylation-deficient Pah1 when the non-mutated phosphorylation sites of the enzyme are presumably in their phosphorylation state (51, 57).

#### Pah1 expression

**Figure 8:**
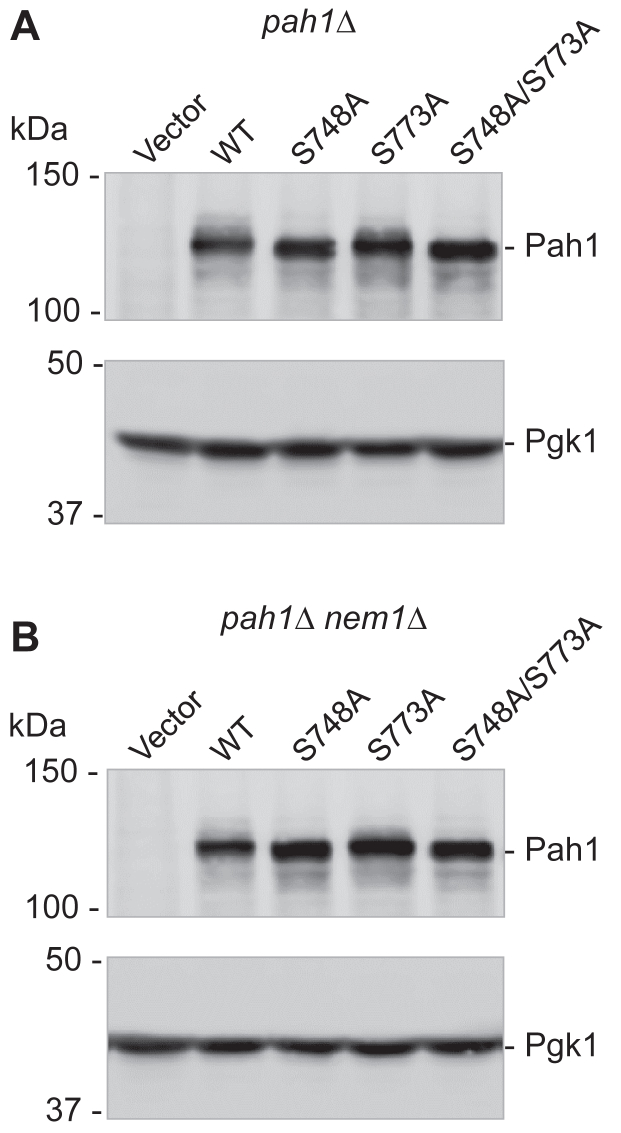
**Expression of the phosphorylation-deficient S748A, S773A, and S748A/S773A mutant forms of Pah1 in *pah1*Δ and *pah1*Δ *nem1*Δ mutant cells.** The *pah1*Δ (*A*) and *pah1*Δ *nem1*Δ (*B*) cells expressing the indicated WT and Hsl1 phosphorylation-deficient S748A, S773A, and S748A/S773A mutant forms of Pah1 were grown at 30 °C to the mid-logarithmic phase in SC-Leu medium. Cell lysates were prepared and equal amounts based on a cell density of A_600_ = 0.2 were subjected to SDS-PAGE using a 10% polyacrylamide gel, followed by immunoblot analysis using anti-Pah1 and anti-Pgk1 (loading control) antibodies. The positions of Pah1 and Pgk1 are indicated with molecular mass standards. The data shown is representative of three independent cultures.

The levels of the WT Pah1 and its phosphorylation-deficient forms were examined from cells in the exponential phase (Fig. 8). This growth phase is when Hsl1 is expressed (Fig. 3) and is expected to act on Pah1. Immunoblot analysis of the cell lysates with anti-Pah1 antibody confirmed that the phosphorylation-deficient variants are expressed in both *pah1*Δ and *pah1*Δ *nem1*Δ genetic backgrounds (Fig. 8, *A* and *B*). In addition, their protein levels were not significantly different form that of WT Pah1 (Fig. 3). Compared with WT Pah1, the S748A variant showed a faster electrophoretic mobility, indicating that the serine phosphorylation affects Pah1 migration in SDS-PAGE (57). The S773A mutant, however, did not show a difference in the electrophoretic mobility.

#### Lipid synthesis

**Figure 9:**
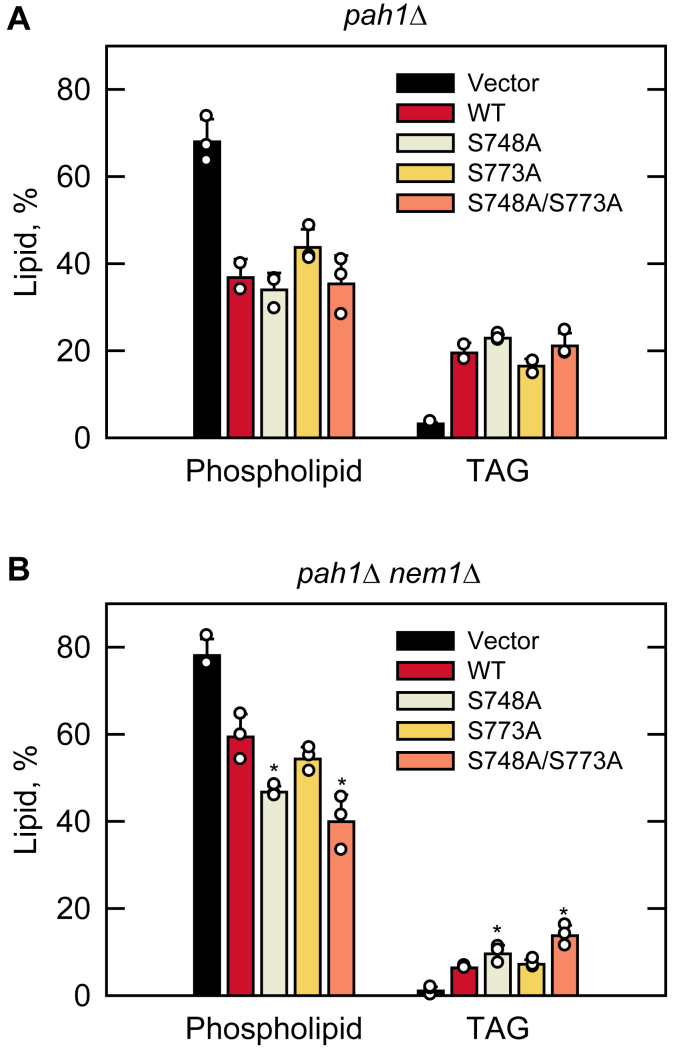
**Hsl1 phosphorylation-deficient S748A and S773A mutations in Pah1 cause a decrease in the synthesis of phospholipids and an increase in TAG synthesis in *pah1*Δ *nem1*Δ mutant cells.** The *pah1*Δ (*A*) and *pah1*Δ *nem1*Δ (*B*) cells expressing the indicated WT and Hsl1 phosphorylation-deficient S748A, S773A, and S748A/S773A mutant forms of Pah1 were grown at 30 °C to the exponential phase in 2 ml of SC-Leu medium. The cells were then incubated with [2-^14^C]acetate (1 μCi/ml) for 20 min. Lipids were extracted, separated by one-dimensional TLC, and subjected to phosphorimaging, followed by ImageQuant analysis. The percentages shown for phospholipids and TAG were normalized to the total ^14^C-labeled chloroform-soluble fraction. The data are means ± S.D. (*error bars*) from three separate experiments. The individual data points are also shown. ∗, *p* < 0.05 *versus* WT.

The *pah1*Δ and *pah1*Δ *nem1*Δ cells expressing the phosphorylation-deficient Pah1 were examined for lipid synthesis in the exponential phase by incubation with [2-^14^C]acetate for 20 min. The incorporation of the radiolabel into each lipid species in the pulse labeling reflects a rate of synthesis (91). As described previously (33), the *pah1*Δ mutant showed a very low level of TAG synthesis (3%) and a resultant high level of phospholipid synthesis (68%) (Fig. 9). The expression of WT Pah1 complemented the *pah1*Δ mutant by increasing its TAG synthesis to the level of 20% but decreasing phospholipid synthesis to 37% (Fig. 9*A*). Similar to the effect of WT Pah1, the phosphorylation-deficient forms S748A and S773A complemented the defect of the *pah1*Δ mutant in lipid synthesis (Fig. 9*A*). In contrast to the expression of WT Pah1 in *pah1*Δ cells, its expression in *pah1*Δ *nem1*Δ cells showed a very weak complementation effect on TAG synthesis (Fig. 9*B*), which emphasizes the requirement of the Nem1-Spo7-mediated dephosphorylation of Pah1 to activate its function in lipid synthesis (57, 92). Compared with WT Pah1, its phosphorylation-deficient forms S748A and S748A/S773A expressed in *pah1*Δ *nem1*Δ cells resulted in a significantly higher rate of TAG synthesis (Fig. 9*B*). For example, the mutant cells expressing the S748A/S773A form exhibited a 117% increase in TAG synthesis and a 33% decrease in phospholipids synthesis (Fig. 9*B*). Interestingly, the S773A mutant alone did not show a significant effect on lipid synthesis. These results indicate that Pah1 deficient in phosphorylation on Ser-748 and Ser-773 partially bypasses the requirement of Nem1-Spo7 for its role in lipid synthesis.

#### Nuclear morphology

**Figure 10:**
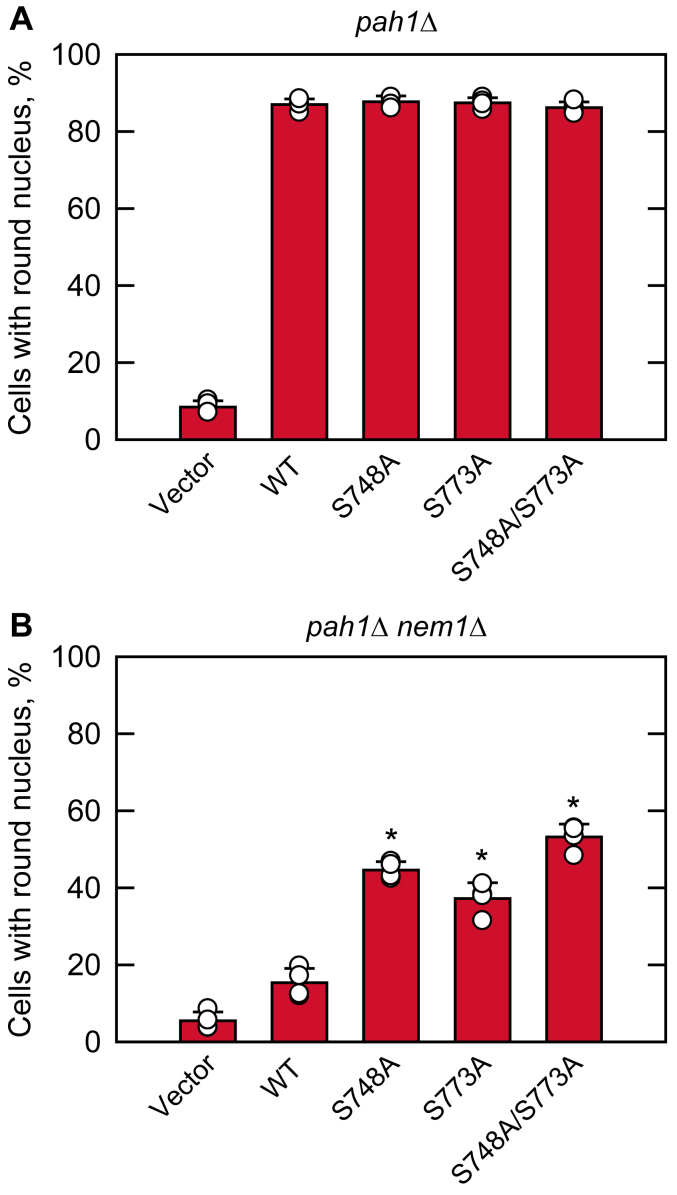
**Hsl1 phosphorylation-deficient S748A and S773A mutations in Pah1 afford complementation of the aberrant nuclear morphology of *pah1*Δ *nem1*Δ mutant cells.** The *pah1*Δ (*A*) and *pah1*Δ *nem1*Δ (*B*) cells expressing the indicated WT and Hsl1 phosphorylation-deficient S748A, S773A, and S748A/S773A mutant forms of Pah1, and expressing the GFP-tagged nuclear/ER membrane marker Sec63 from plasmid YCplac33-*SEC63-GFP* were grown at 30 °C in SC-Leu-Ura medium to the exponential phase of growth. The percentage of cells with round nuclear/ER morphology was determined from ≥4 fields of views (≥200 cells). The data are averages ± S.D. (*error bars*). The individual data points are also shown. ∗*p* < 0.05 *versus* round nucleus of WT cells.

The mutants defective in Pah1 and/or Nem1-Spo7 display irregularly enlarged nuclei because of the increase in phospholipid synthesis and the aberrant expansion of the nuclear/ER membrane (30, 34, 35, 41, 60, 67) (Fig. 10*A* and *B*, *vector control*). The complementation of this phenotype was scored by fluorescence microscopy of the ER membrane marker Sec63-GFP (Fig. 10). Almost 90% of *pah1*Δ cells expressing WT or the phosphorylation-deficient mutant forms of Pah1 had a round nucleus (Fig. 10*A*). As described previously for the Nem1-Spo7 complex-deficient cells (60, 67, 93), only 15% of *pah1*Δ *nem1*Δ cells expressing WT Pah1 exhibited a round nucleus (Fig. 10*B*). In contrast, the phosphorylation-deficient forms expressed in *pah1*Δ *nem1*Δ cells had significant effects on the formation of a round nucleus (Fig. 10*B*). Compared with the number of cells expressing WT Pah1 (15%), the numbers of cells expressing the S748A, S773A, and S748A/S773A mutants increased 44, 37, and 53%, respectively.

## Discussion

Phosphorylation of *S. cerevisiae* Pah1 plays an important role in its subcellular location, PAP activity, and stability/degradation (49). Here, we established that Pah1 is a *bona fide* substrate of Hsl1, a protein kinase associated with bud formation (70, 71, 72), and that phosphorylation of Pah1 by Hsl1 promoted phospholipid synthesis in the exponential phase of growth. Using Pah1 as substrate, Hsl1 activity followed zero order kinetics; the enzyme reaction was dependent on the amount of Hsl1 and time. The *K*_m_ values of Hsl1 for Pah1 and ATP were in the low μmolar range and similar to those of other protein kinases acting on the protein substrate (Table 1). Ser-748 and Ser-773 were identified as the major sites of Pah1 phosphorylation with the latter site accounting for 76% of the phosphorylation. The serine phosphorylations together caused a 5-fold reduction in PAP activity, which is similar to the inhibitory effect of Pah1 phosphorylation by Pho85, a cyclin-dependent protein kinase that phosphorylates Ser-748 and 6 additional sites (50) (Fig. 2, Table 1). In contrast to Hsl1 and Pho85, the casein kinase I phosphorylation of Pah1 on Ser-748 stimulates its PAP activity (55). This stimulatory effect might be explained by Pah1 phosphorylation on the non-overlapping sites that regulate PAP activity in a manner opposite to that of Hsl1 (55) (Fig. 2, Table 1). Phosphorylation of Pah1 by protein kinases A (52) and C (53), both of which share Ser-773 as a phosphorylation site with Hsl1, causes a moderate inhibition of PAP activity, and the contribution of Ser-773 to the overall inhibition by either protein kinase is very small (52, 53). Accordingly, we speculate that the inhibitory effect of Hsl1 on Pah1 activity is mainly due to its phosphorylation on Ser-748, which accounts for only 24% of the total phosphorylation. The variation between the effects of phosphorylation by the protein kinases, despite acting on the common target sites, highlights the complex nature of the phosphorylation-mediated regulation of Pah1 function. That multiple protein kinases target the same residue emphasizes the importance of maintaining its phosphorylation when they are active at different times and growth conditions.

**Table 1:** Kinetic properties of protein kinases that phosphorylate Pah1

Protein kinase	Pah1	ATP	Pah1 phosphorylation sites	Reference
	*K*_*m*_	*K*_*m*_		
	*μM*	*μM*		
Hsl1	0.39	31	Ser-748, Ser-773	This study
Casein kinase I	0.21	2.4	Ser-114, Ser-475, Ser-511, Ser-602, Ser-677, Ser-748, Ser-774	(55)
Casein kinase II	0.23	5.5	Thr-170, Ser-250, Ser-313, Ser-705, Ser-814, Ser-818	(54)
Cdc28	0.21	5.8	Ser-602, Thr-723, Ser-744	(51)
Pho85	0.25	3.7	Ser-110, Ser-114, Ser-168, Ser-602, Thr-723, Ser-744, Ser-748	(50)
Protein kinase A	0.44	4.4	Ser-10, Ser-677, Ser-773, Ser-774, Ser-788	(52)
Protein kinase C	0.75	4.5	Ser-677, Ser-769, Ser-773, Ser-788	(53)
Rim11	0.40	30	Ser-12, Thr-163, Thr-164, Thr-522, Ser-602, Ser-818	(56)

In the pulse labeling of lipid synthesis, the lack of Ser-748 phosphorylation alone, and in combination with the loss of Ser-773 phosphorylation, caused a significant increase in TAG synthesis with a corresponding decrease in phospholipid synthesis. These mutational effects, which were notable in the *nem1*Δ mutant, indicate the requirement of Pah1 phosphorylation at specific sites for its regulation by the Nem1-Spo7 complex (50, 51, 57, 61). The S773A mutation was not as effective as the S748A mutation in the absence of the Nem1-Spo7 complex, but the combined mutations had a stronger effect on Pah1 function (*e.g.*, TAG synthesis and nuclear morphology). The mutational effects of S748A and S773A, which bypass the requirement of Nem1-Spo7 for Pah1 function (57, 60, 61), are similar to those shown by Pah1-7A (50, 51), which contains non-phosphorylable alanine residues in place of seven residues including Ser-748 phosphorylated by Pho85 (50).

Although Pah1 mutants that lack the target sites of Hsl1 or Pho85 exhibit a gain-of-function phenotype in the absence of Nem1-Spo7, they show a difference with respect to the role of phosphorylation in protein stability/degradation. In general, phosphorylated Pah1 is protected against its degradation by the 20S proteasome, whereas the enzyme dephosphorylated by Nem1-Spo7 is susceptible to proteasomal degradation (59). The phosphorylation sites of Pah1 that are most affected by proteasomal degradation are the seven sites targeted by Pho85 (50, 59). For example, when Pah1-7A is expressed in *pah1*Δ and *pah1*Δ *nem1*Δ cells, its abundance is greatly reduced when compared with that of the WT protein (50). In striking contrast, the protein levels of the phosphorylation-deficient S748A and S773A mutants, in both genetic backgrounds, were similar to that of the WT control. This indicates that the sites of Pah1 phosphorylated by Hsl1 do not play a key role in controlling the protein stability.

Hsl1 is also known to engage in genetic interactions with other members of the Pah1-regulating network of protein kinases. Hsl1 exhibits a genetic interaction with Slt2, a MAP kinase that is activated by protein kinase C in a signaling cascade that is active when the morphogenesis checkpoint is triggered (94). Protein kinase C activity is dependent on DAG produced by Pah1 (95), highlighting the importance of Pah1 during exponential growth. Hsl1 also has a functional relationship with Cdc28. A major function of Hsl1 is to downregulate Swe1, a Cdc28 inhibitor, suggesting that Hsl1 and Cdc28 are active at the same time and therefore may phosphorylate Pah1 at the same time (96). Swe1 is also predicted to phosphorylate Pah1 (69), raising a possibility that its phosphorylation by Swe1 has an opposing effect on Pah1 when compared to its phosphorylation by Hsl1 and Cdc28. The condition in which Swe1-dependent phosphorylation of Pah1 stimulates its PAP activity would prevent the biogenesis of membrane phospholipids while Cdc28 is inhibited and cell growth is arrested.

The *in vivo* phosphorylation of Pah1 by Hsl1 is also likely to be affected by other protein kinases that phosphorylate Hsl1. Like Pah1, Hsl1 is subject to regulation by multiple phosphorylation (97). The premise that the protein kinases acting on Pah1 may also phosphorylate each other was previously raised for Rim11, which appears to be phosphorylated by Pho85 in reactions where both protein kinases are present along with the substrate Pah1 (56). Protein kinases known to phosphorylate Hsl1 include Cdc28, a protein kinase for which Pah1 phosphorylation has been characterized (51), and Mck1, a glycogen synthase kinase 3β homolog that is predicted to phosphorylate Pah1 (69, 79, 98).

In addition to the Pah1-targeting protein kinases, Hsl1 is phosphorylated by the protein kinase Elm1; phosphorylation at the activation loop Thr-273 is required for Hsl1 protein kinase activity (87). Interestingly, the activation loop is found in at least two other protein kinases, the AMP-activated kinase Snf1 and the spindle position checkpoint protein kinase Kin4, both of which are predicted to phosphorylate Pah1 (69, 99, 100). This raises a possibility that Elm1 has a larger role in lipid metabolism that is mediated by its activation of Pah1-targeting protein kinases, which would be consistent with a lipidomics study showing that glycerophospholipid levels are altered in the absence of Elm1 (101). Similar to Hsl1, both Snf1 and Kin4 have functions related to cellular proliferation during the exponential phase of growth. Further studies into Pah1 phosphorylation by Kin4 and Snf1 will elucidate the mechanism of Elm1-dependent regulation of lipid metabolism and may give insight into whether these Elm1-regulated protein kinases have a synergistic effect on Pah1 phosphorylation during cell proliferation.

The mammalian homolog of *S. cerevisiae* Pah1 is known as lipin (30). The three spliced variants of lipin 1 (α, β, and γ), along with the lipin 2 and 3 isoforms are PAP enzymes (30, 102, 103). The critical roles that PAP activity plays in humans and mice are typified by assorted abnormalities (*e.g.*, lipodystrophy, insulin resistance, peripheral neuropathy, rhabdomyolysis) that result from the loss of the lipin 1 enzyme (104, 105, 106, 107, 108, 109). In the mouse model, excess lipin 1 results in the obese phenotype (104, 110). Like Pah1, lipins 1 and 2 are subject to multiple phosphorylation (111, 112, 113, 114, 115), and for at least lipin 1, the enzyme is dephosphorylated by the mammalian counter part of the Nem1-Spo7 protein phosphatase complex known as CTDNEP1-NEP1-R1 (108, 116, 117, 118, 119). As in *S. cerevisiae*, the phosphorylation state of lipin 1 governs its subcellular localization (111, 112, 113, 120, 121). Mammalian homologs of Hsl1 are BRSK1 and 2. These serine/threonine protein kinases are specifically expressed in the brain and play key roles in neuronal development (122, 123). Whether they phosphorylate and regulate lipin activity and/or its localization is unclear, but the conservation of the *S. cerevisiae* Nem1-Spo7/Pah1 phosphatase cascade in mammalian cells suggests an important avenue of investigation.

## Experimental procedures

### Reagents

Avanti Polar Lipids and Analtech, respectively, supplied lipids and silica gel GHL TLC plates. DNA size ladders, molecular mass protein standards, and reagents for electrophoresis and Western blotting were from Bio-Rad. BioSynthesis, Inc prepared the rabbit anti-Pah1 antibody directed against the sequence TSIDKEFKKLSVSKAGA (residues 778–794) (51). Cayman Chemical was the supplier of protease inhibitors leupeptin and pepstatin. Carrier DNA for yeast transformations was from Clontech. Growth media were purchased from Difco Laboratories. InstantBlue Coomassie stain was from Expedeon. GE Healthcare was the supplier of Q-Sepharose, IgG-Sepharose, polyvinylidene difluoride membrane, and the chemifluorescence Western blotting detection kit. Mouse anti-Pgk1 antibody (product number: 459250; lot number: E1161) was from Invitrogen. MilliporeSigma supplied ATP, bovine serum albumin, Ponceau S stain, protease and phosphatase inhibitors, Triton X-100, rabbit anti-calmodulin binding protein epitope tag antibody (product no. 07-482, lot no. 3467112), and alkaline phosphatase-conjugated goat anti-mouse IgG antibody (product no. A3562; lot no. SLBG1482V). National Diagnostics was the supplier of scintillation counting supplies. Q5 site-directed mutagenesis kit and other reagents for DNA manipulations were from New England Biolabs. Radiochemicals were purchased from PerkinElmer Life Sciences. DNA gel extraction and plasmid purification kits and the nickel-nitrilotriacetic acid agarose resin were from Qiagen. Thermo Fisher Scientific was the source of Pierce mass spectrometry grade proteases and strong anion exchange spin columns, alkaline phosphatase-conjugated goat anti-rabbit IgG antibody (product no. 31340, lot number: NJ178812), and *S. cerevisiae* strain BY4741-*HSL1*-TAP. All other chemicals were reagent grade.

### *Strains, plasmids, and growth* conditions

The strains and plasmids used in this study are listed in Table 2. *S. cerevisiae* strain BY4741-*HSL1*-TAP was used for the purification of TAP-tagged Hsl1. Strains SS1026 and SS1132 were used for the expression of the WT and phosphorylation-deficient mutant forms of Pah1 in the presence and absence of the Nem1-Spo7 protein phosphatase complex, respectively. *E. coli* strains DH5α and BL21(DE3)pLysS were used for the propagation of plasmids and heterologous expression of His_6_-tagged Pah1, respectively. Plasmids containing *PAH1* (S748A, S773A, and S748A/S773A) were generated from pGH315 using the Q5 Site-directed mutagenesis kit. Standard methods were used for the isolation of plasmid DNA, for digestion and ligation of DNA, and for PCR amplification of DNA (124, 125, 126). Plasmid transformations of *E*. *coli* (125) and *S. cerevisiae* (127) were performed by standard methods. DNA constructs were confirmed by PCR analysis and DNA sequencing.

**Table 2:** Strains and plasmids used in this study

Strain or plasmid	Genotype or relevant characteristics	Source or Reference
Strain		
*S*. *cerevisiae*		
BY4741-*HSL1*-TAP	TAP-tagged HSL1 strain	Thermo Fisher Scientific
RS453	MATa ade2-1 his3-11,15 leu2-3112 trp1-1 ura3-52	(148)
SS1026	*pah1*Δ*::TRP1* derivative of RS453	(34)
SS1132	*pah1*Δ*::TRP1 nem1*Δ*::HIS3* derivative of RS453	(51)
*E.coli*		
DH5α	F^-^ ϕ80d*lacZ*ΔΜ15Δ (*lacZYA*-*argF*)U169 *deoR rec*A1 *end*A1 *hsd*R17 (*r*_k_^-^*m*_*k*_^+^) *pho*A *sup*E44 λ^−^*thi-*1 *gyr*A96 *rel*A1	(125)
BL21 (DE3)pLysS	F^-^*ompT hsdS*_*B*_ (*r*_*B*_^-^*m*_*B*_^-^) *gal dcm* (DE3) pLysS	Novagen
Plasmid		
pET-15b	*E. coli* expression vector with N-terminal His_6_-tag fusion	Novagen
pGH313	*PAH1* coding sequence inserted into pET-15b	(30)
pRS415	Single-copy number *E. coli*/yeast shuttle vector with *LEU2*	(149)
pGH315	*PAH1* inserted into pRS415	(92)
pGH315-S748A	*PAH1* (S748A) derivative of pGH315	(50)
pGH315-S773A	*PAH1* (S773A) derivative of pGH315	(52)
pGH315-S748A/S773A	*PAH1* (S748A/S773A) derivative of pGH315	This study
YCplac33-SEC63-GFP	*SEC63-GFP* fusion inserted into the *CEN/URA3* vector	(44)

*S. cerevisiae* cells were cultured using standard methods (124, 125). Solid media plates contained 2 or 1.5% agar for the growth of *S. cerevisiae* or *E. coli*, respectively. Cells expressing TAP-tagged Hsl1 were grown at 30 ^°^C in 2 L YEPD (1% yeast extract, 2% peptone, 2% glucose) medium. Growth in liquid medium was monitored by absorbance at 600 nm (A_600_) using a spectrophotometer. *S. cerevisiae* cells carrying a plasmid were grown at 30 °C in a synthetic drop-out medium, which lacks a specific amino acid from a synthetic complete (SC) medium for the plasmid selection. The *E*. *coli* cells were grown at 37 °C in lysogeny broth (LB) medium (1% tryptone, 0.5% yeast extract, 1% NaCl, pH 7.0); ampicillin (100 μg/ml) was added to select for cells carrying plasmids. For His_6_-tagged Pah1 expression, the bacterial cells harboring plasmid pGH313 were grown to A_600_ ∼0.5 at room temperature in 500 ml of LB medium containing ampicillin (100 μg/ml) and chloramphenicol (34 μg/ml); expression was induced for 1 h with 1 mM isopropyl-*β*-D-thiogalactoside (30).

### Preparation of cell lysates and enzyme isolations

Yeast cells were collected by centrifugation at 1500*g* for 5 min. The cells were washed with water and boiled in lysis buffer containing 50 mM Tris-HCl (pH 7.5), 10% glycerol, 10 mM 2-mercaptoethanol, 1 mM EDTA, 0.5 mM phenylmethylsulfonyl fluoride, 1 mM benzamidine, 5 μg/ml aprotinin, 5 μg/ml leupeptin, 5 μg/ml pepstatin, and 8% SDS. The cell lysates were directly used for SDS-PAGE and immunoblotting. All procedures for enzyme purification were conducted at 4 ^°^C. His_6_-tagged Pah1 expressed in *E. coli* was purified to near homogeneity from cell extracts by affinity chromatography with nickel-nitrilotriacetic acid-agarose (30), followed by ion exchange chromatography with Q-Sepharose (61). TAP-tagged Hsl1 was partially purified by affinity chromatography with IgG-Sepharose (73, 128). *S. cerevisiae* cells expressing TAP-tagged Hsl1 were harvested, and the cell pellet was resuspended in 50 mM Tris-HCl (pH 8.0) buffer containing 150 mM NaCl, 1 mM EDTA, and Roche EDTA-free protease inhibitors. The cells were then lysed with glass beads using a Mini-Beadbeater-16 (5 repeats of 1-min burst with 2-min cooling between bursts). The cell lysate was centrifuged at 1500*g* for 10 min, and the supernatant was mixed with an equal volume of 50 mM Tris-HCl (pH 8.0) buffer containing 150 mM NaCl, 1 mM EDTA, Roche EDTA-free protease inhibitors, and 2% Triton X-100 and centrifuged at 100,000*g* for 1 h. The supernatant was applied to a 0.5 ml IgG-Sepharose column equilibrated with 50 mM Tris-HCl (pH 8.0) buffer containing 150 mM NaCl, 0.5 mM EDTA, and 0.1% Triton X-100. The column was washed with the equilibration buffer with 10 mM Tris-HCl (pH 8.0) to lower buffer capacity, and fusion protein was eluted with 50 mM glycine (pH 3.0) and 0.1% Triton X-100 (55) and neutralized by the addition of 0.2 volume of 1M Tris-HCl (pH 8.0). The final protein concentration of the purified enzyme was 1 μg/ml. The purified enzyme preparation was stored at −80 °C after the addition of glycerol to a final concentration of 10%.

### Protein kinase assay

Hsl1 protein kinase activity was measured at 30 ^°^C by following the incorporation of radioactive phosphate from [γ-^32^P]ATP into Pah1 in a total volume of 20 μl as described previously (51). The reaction mixture contained 50 mM Tris-HCl (pH 7.5), 10 mM MgCl_2_, 100 μM [γ-^32^P]ATP (∼3000 cpm/pmol), 0.25 μM Pah1, 2 mM dithiothreitol, and the indicated amount of Hsl1. The phosphorylation reaction was terminated by the addition of 5 μl 5x Laemmli sample buffer, followed by SDS-PAGE (129) to resolve the ^32^P-labeled Pah1 from radioactive ATP. The phosphorylated Pah1 was visualized by phosphorimaging using a Storm 860 Molecular Imager (GE Healthcare) and the extent of phosphorylation was quantified by ImageQuant software.

### Identification of Hsl1 peptide sequences and phosphorylation site analysis of Pah1 by LC-MS/MS

To confirm the identity of TAP-tagged Hsl1, the fusion protein contained within an SDS polyacrylamide gel slice was digested with trypsin at 37 °C followed by the analysis of the digest by LC-MS/MS (89) (Table S1). The amino acid residues of Pah1 phosphorylated by the Hsl1 protein kinase were analyzed by LC-MS/MS. The proteolytic digestion of the Hsl1-phosphorylated Pah1 in polyacrylamide gel slices, analysis of peptide fragments by LC-MS/MS, and database analysis were performed as described previously (89) (Table S1) at the Center for Integrative Proteomics Research at Rutgers University. The raw data and database results for the peptide analyses of Pah1 and Hsl1 are deposited in the MassIVE repository (https://massive.ucsd.edu/ProteoSAFe/static/massive.jsp) with the accession number MSV000095027.

### SDS-PAGE and Western blot analysis

Standard procedures were used for SDS-PAGE (129) and Western blotting (130, 131). The samples for Western blotting were normalized to total protein loading. Protein transfer from polyacrylamide gels to PVDF membranes was monitored by staining with Ponceau S. Rabbit anti-calmodulin binding peptide, rabbit anti-Pah1, and mouse anti-Pgk1 antibodies were used at a final concentration of 2 μg/ml. A dilution of 1:5000 was used with the secondary goat anti-rabbit IgG antibody and goat anti-mouse IgG antibody that is conjugated with alkaline phosphatase. The enhanced chemifluorescence immunoblotting substrate was used to detect immune complexes. Fluorimaging with a Storm 865 Molecular Imager was used to visualize fluorescence signals from immunoblots; image intensities were analyzed by ImageQuant TL software (GE Healthcare). A standard curve ensured that the immunoblot signals were in the linear range of detection.

### PAP assay

PAP activity was measured by following the release of water-soluble ^32^P_i_ from chloroform-soluble [^32^P]PA (10,000 cpm/nmol) using the Triton X-100/PA mixed micellar assay as described by Carman and Lin (132). The standard reaction mixture contained 50 mM Tris-HCl (pH 7.5), 1 mM MgCl_2_, 0.2 mM PA, 2 mM Triton X-100, and enzyme protein in a total volume of 100 μl. The surface concentrations of PA (mol %) were obtained by maintaining the molar concentration of PA at 0.2 mM and varying the molar concentrations of Triton X-100 (132). Enzyme assays were performed in triplicate and all reactions were linear with time and protein concentration. [^32^P]PA was enzymatically produced from DAG by DAG kinase with [γ-^32^P]ATP (132).

### Protein determination

The protein-dye binding assay of Bradford (133) was used to estimate protein concentration; bovine serum albumin was used as the standard.

### Lipid labeling and analysis

*S. cerevisiae* cells were pulse-labeled with [2-^14^C]acetate for 20 min (33, 134). Lipids were extracted from cells (135, 136) and resolved by one-dimensional TLC on silica gel plates using the solvent system hexane/diethyl either/glacial acetic acid (40:10:1, v/v) (137). The resolved lipids were visualized by phosphorimaging with a Storm 860 Molecular Imager (GE Healthcare) and quantified by ImageQuant software using a standard curve of [2-^14^C]acetate. The position of radiolabeled lipids on the TLC plate was confirmed by comparison with the migration of authentic standards visualized by staining with iodine vapor.

### Microscopic analysis of nuclear morphology

Nuclear/ER morphology was examined by fluorescence microscopy of cells expressing the *SEC63*-*GFP* plasmid (44). The percentage of cells with a round nucleus was scored from ≥4 fields of view (≥200 cells). The microscope used to image the cells was a Nikon Eclipse Ni-U microscope with the EGFP/FITC/Cy2/AlexaFluor 488 filter and fields of view were recorded by a DS-Qi2 camera. Image analysis was performed with NIS-Elements BR software.

### Data analysis

The statistical analysis of data was determined with Microsoft Excel software. The *p* values < 0.05 were taken as a significant difference. The enzyme kinetics module of SigmaPlot software was used to analyze kinetic data.

## Data availability

Raw MS phosphorylation data and database search results for Pah1 and Hsl1 analysis data are deposited in the MassIVE repository (accession number MSV000095027). All other data are contained within the manuscript or the supporting information.

## Supporting information

This article contains supporting information.

## Conflict of interest

The authors declare that they have no conflicts of interest with the contents of this article.
